# Epigenetic age analysis of children who seem to evade aging

**DOI:** 10.18632/aging.100744

**Published:** 2015-05-15

**Authors:** Richard F. Walker, Jia Sophie Liu, Brock A. Peters, Beate R. Ritz, Timothy Wu, Roel A. Ophoff, Steve Horvath

**Affiliations:** ^1^ Physician's Scientific and Regulatory Services, Inc., Indian Rocks Beach, FL 33785, USA; ^2^ Department of Research, Complete Genomics Inc. Mountain View CA94043 USA, BGI-Shenzhen, Shenzhen 518083, China; ^3^ Epidemiology, School of Public Health, University of California Los Angeles, Los Angeles, CA 90095, USA; ^4^ Human Genetics, David Geffen School of Medicine, University of California Los Angeles, Los Angeles, CA 90095, USA; ^5^ UCLA Center for Neurobehavioral Genetics, Semel Institute for Neuroscience and Human Behavior, University of California Los Angeles, Los Angeles, CA 90095, USA; ^6^ Biostatistics, School of Public Health, University of California Los Angeles, Los Angeles, CA 90095, USA

**Keywords:** slow aging, epigenetic clock, DNA methylation, biomarker of aging, syndrome X

## Abstract

We previously reported the unusual case of a teenage girl stricken with multifocal developmental dysfunctions whose physical development was dramatically delayed resulting in her appearing to be a toddler or at best a preschooler, even unto the occasion of her death at the age of 20 years. Her life-long physician felt that the disorder was unique in the world and that future treatments for age-related diseases might emerge from its study. The objectives of our research were to determine if other such cases exist, and if so, whether aging is actually slowed. Of seven children characterized by dramatically slow developmental rates, five also had associated disorders displayed by the first case. All of the identified subjects were female. To objectively measure the age of blood tissue from these subjects, we used a highly accurate biomarker of aging known as “epigenetic clock” based on DNA methylation levels. No statistically significant differences in chronological and epigenetic ages were detected in any of the newly discovered cases.

Our study shows that a) there are multiple children who maintain the façade of persistent toddler-like features while aging from birth to young adulthood and b) blood tissue from these cases is not younger than expected.

## INTRODUCTION

**Figure 1:**
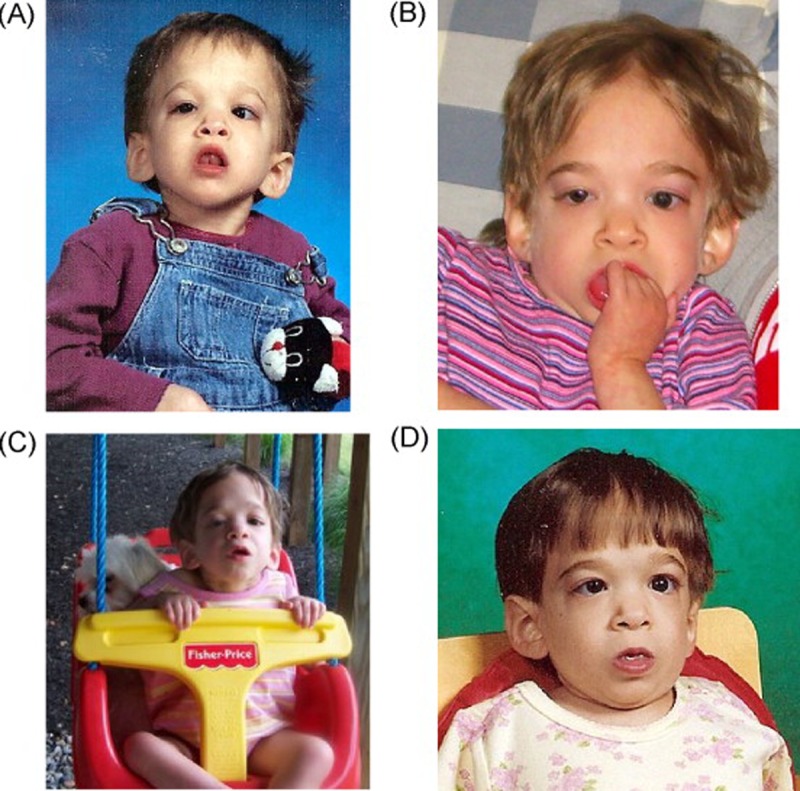
Photographs of the first case at various ages (**A**) 5 years old, (**B**) 10 years old, (**C**) 11 years old, (**D**) 15 years old. Note that only minimal changes in physical appearance can be observed during the transition from childhood to adolescence. The case was not part of this study. Credits: Reproduced with kind permission from the publisher of [1].

We previously reported the unusual case of a teenage girl stricken with multifocal developmental dysfunctions including bilateral hip dislocations, abnormal brain development, tracheomalacia, gastro-intestinal disorders and dysmorphic features [1, 2]. In addition to these problems, her physical and functional development was dramatically delayed resulting in her appearing to be a toddler or at best a preschooler, even unto the occasion of her death at the age of 20 years (Figure 1). Her developmental rate, albeit significantly delayed, continued nonetheless. But the extremely slow rate of change gave the impression that she was not aging at all.

As the result of her persistent “toddler-like” appearance, she received extensive notoriety from the media, and was featured as the “girl who doesn't age” in press articles and television broadcasts [3-6]. Dr. Lawrence Pakula, the pediatrician who cared for her from birth described his patient's strange affliction, that did not fit any disease category, as an “unknown syndrome” later to be called “syndrome X” [5]. While the word syndrome accurately describes a group of symptoms that collectively indicate or characterize a disease, Pakula was also quoted as saying that “there's no one else like her in the world”. While most of the individual defects she experienced are not uncommon in many children, it was her retaining toddler-like features while aging from birth to young adulthood that made the case particularly unusual. Even children with growth retardation or failure to thrive exhibit maturation of facial and other physical/functional features with passage of time, indicating that their developmental program is still functional. In contrast, the peculiar trait of the first case suggested that her rate of aging was dramatically delayed or even arrested. If so, then perhaps an etiological understanding of her pathology might lead to novel treatments for age related diseases [2, 6].

The objectives of this study were two-fold. The first was to determine if other such cases of syndrome X actually exist and thus might represent a novel syndrome. Then, because the case's appearance remained that of a toddler despite the passage of time, our second objective was to determine if there was any evidence that the arrested development in such children is linked to a slowing down of aging at the molecular level.

Objectively measuring the age of tissue such as blood poses a significant methodological and conceptual challenge since it is debatable whether known causes of cellular senescence such as telomere length represent tissue, much less organismal aging [7-10]. We recently developed a biomarker of aging called epigenetic clock that is based upon DNA methylation (DNAm) levels [11]. The epigenetic clock is currently the preferred biomarker of organismal aging because a) it is more strongly correlated with chronological age than previous biomarkers including telomere length [12, 13], b) it is prognostic of all-cause mortality in later life [14], and c) it correlates with measures of physical and mental fitness in older age [15]. The utility of the method has been demonstrated in recent case studies, e.g. a) obesity accelerates epigenetic aging in liver tissue [13], b) trisomy 21 (Down syndrome) accelerates epigenetic aging [16], c) HIV infection accelerates epigenetic age [22], and d) the cerebellum ages slowly [23].

## RESULTS

Here we applied the epigenetic clock method to assess the epigenetic age of blood tissue from 7 syndrome X cases and n=106 controls (total n=113) as described in the following.

### Selection of cases and controls

The first case who passed away at the age of 20 due to respiratory complications [17], was not part of this epigenetic study. To achieve our study objectives, we first needed to determine whether the clinical condition of the first case was indeed unique in the world. Many families with children suffering from developmental delay and related complications responded to the broadcasts covering the first case seeking help from Dr. Walker. The parents of subjects described in this study volunteered to participate because one of their children was diagnosed with clinical symptoms comparable to those of our first case. Ethical review of this study was conducted and approved by the UCLA IRB on April 30, 2014 (IRB#14-000140, “Genetic and epigenetic study of children with severely delayed development or aging”).

Each family provided detailed information to allow verification that their child's condition was in fact, similar to those reported for the first case. As expected, the majority of respondents had children with developmental delay easily attributable to known factors. These families were excluded from the study. On the other hand, several subjects were identified as potential candidates for further study after careful review of their medical and family records (such as written descriptions, diaries, and pictures). Like the first case, all failed to grow even when provided with hormone replacement or hypercaloric diets and appeared to be significantly younger than their chronological ages. They were also afflicted with similar neurological, gastrointestinal, cardiac, and orthopedic problems suggesting that their clinical conditions might actually represent a novel syndrome.

The following criteria were used for inclusion in the study. Except for the first two which were required, all subjects met a majority of the remaining criteria although differing to some degree in severity.

1. Physical appearance and developmental characteristics of an infant/toddler despite chronological ages normally associated with more mature features.
2. Retarded growth rate of height standard deviation score (SDS) more than 3 SDS below the mean for chronological age and sex; failure of hormonal and/or dietary interventions intended to overcome the deficit.
3. Dysmorphic features,
4. Club feet and hip dislocations,
5. Brain abnormalities including but not limited to agenesis of corpus callosum, pachygyria, lissencephaly and polymicrogyria,
6. Minimal cognitive development, lack of speech, visual and/or hearing defects,
7. Laryngotracheomalacia and reactive airway disease,
8. Gastrointestinal developmental disorders and swallowing dysfunction often requiring G-tube for feeding.

Apart from seven families with potentially affected children, we also included five “control” families with children whose ages were comparable to those of the study subjects. We also included 62 unrelated healthy control subjects aged between 35 and 61.

**Figure 2:**
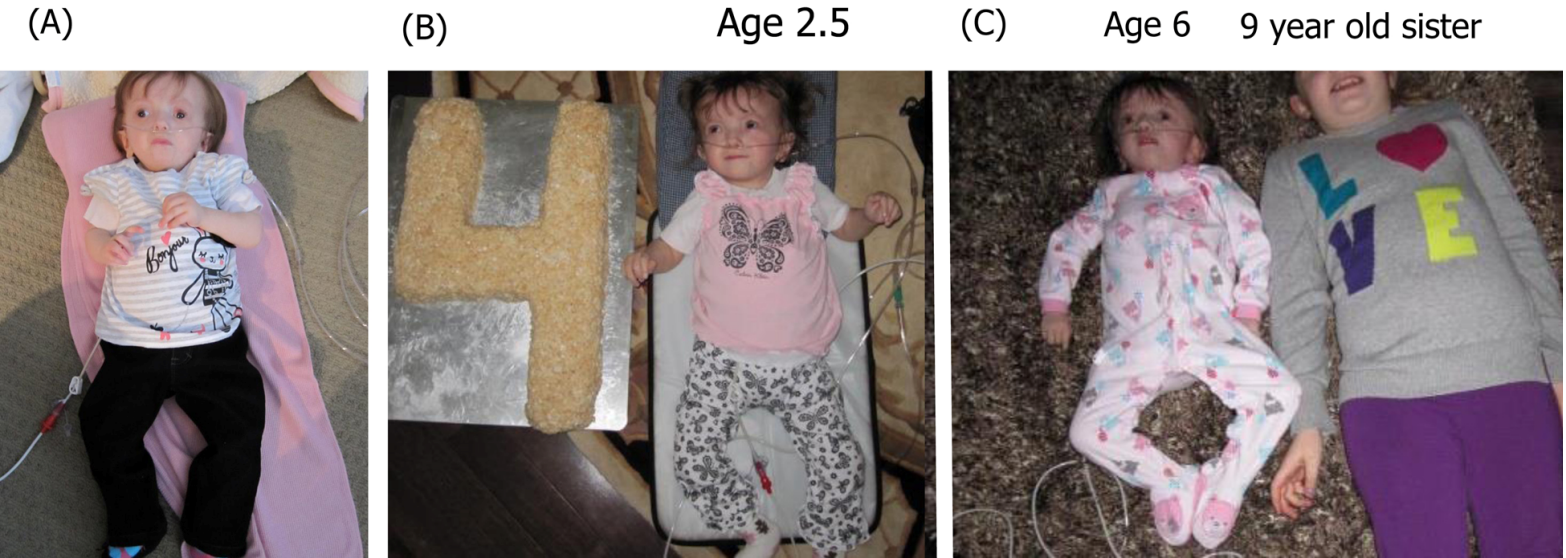
Photographs of one of the cases in the current study at various ages (**A**) 2.5 years old, (**B**) 4 years old, (**C**) 6 years old next to her 9 year old sister. Note that only minimal changes in physical appearance can be observed.

Thirteen of twenty suspected syndrome X children did not meet the stringent inclusion criteria because they exhibited growth problems of known etiology or did not present as toddlers or pre-schoolers at an inappropriate age. However, five suspected cases met the stringent inclusion criteria and presented no evidence of chromosomal abnormalities according to a karyotype and microarray-based comparative genomic hybridiza-tion (CGH) analysis. We consider these five female subjects aged 2.3, 2.5, 5.8, 9.6, and 11.3 years as representing “pure” cases of syndrome X. Apparent lack of physical maturation in one of these children is shown in Figure 2. While two of the 20 suspected cases did not meet all of the syndrome X criteria, we studied them as well because they displayed strikingly delayed aging, atypical of their diagnosed syndromes. These included a 5.5 year old girl with Turner syndrome (Ring-X) and a 25.3 year old woman with trisomy 21 (Down syndrome). The fact that the Down syndrome (DS) case failed to develop/age in a typical fashion is interesting since DS subjects arguably exhibit several clinical manifestations of accelerated aging including early onset Alzheimer's disease [18-20].

### Epigenetic age analysis

**Figure 3:**
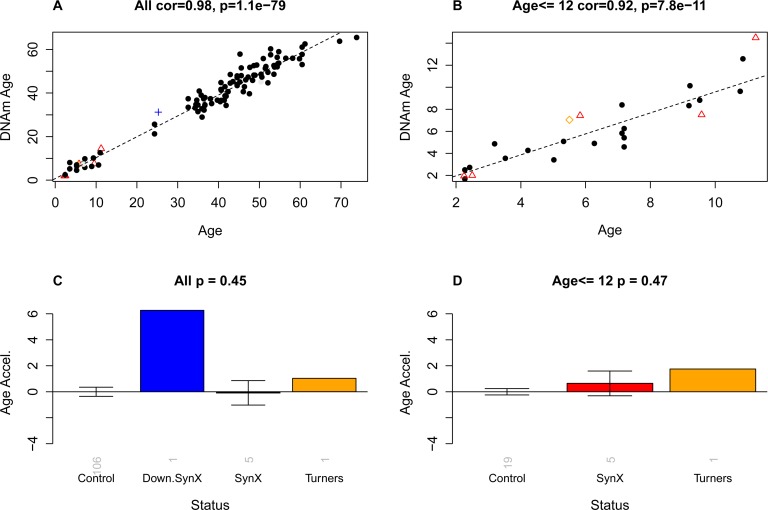
Epigenetic age versus chronological age and disease status The first row (**A,B**) shows scatter plots between chronological age (x-axis) and DNAm age (y-axis) in (**A**) all subjects and (**B**) in subjects younger than or equal to 12. Red triangles indicate the pure syndrome X subjects. The blue cross and the orange diamond correspond to the Down syndrome and Turner syndrome case, respectively. The dashed black line corresponds to a regression line through control subjects. Note that the red triangles do not lie below the regression line, i.e. there is no evidence that these subjects are younger than expected. For each subject, a measure of epigenetic age acceleration was defined as vertical distance between the corresponding point and the regression line in the scatter plot. (**C,D**) Mean epigenetic age acceleration (y-axis) versus disease status in (**C**) all subjects and (**D**) younger subjects, respectively. By definition, the mean age acceleration measure in controls is zero. The title of the bar plots also reports a p-value from a non-parametric group comparison test (Kruskal Wallis test). Each bar plot depicts the mean value and 1 standard error.

Epigenetic age was calculated using the epigenetic clock method/software described in Methods and in [11]. DNAm age of blood was highly correlated with chronological age in all subjects (r=0.98; Figure 3A) and in subjects younger than 12 years (r=0.92; Figure 3B), which validates the high accuracy of this biomarker of aging. Surprisingly, we did not find any evidence that blood tissue from the five pure syndrome X subjects was younger than expected based on their chronological age (Figure 3C, D). The mean DNAm age of the five pure syndrome X subjects was 6.7 years (standard error=1.0) which is not significantly different from the mean chronological age of 6.3 years (standard error=1.8). Notably, the oldest pure syndrome X case had a DNAm age of 14.5 years which was 3.2 years older than her true chronological age. The DNAm age of the case with Turner syndrome was 1.5 years older than her chronological age (5.5) which was well within the margin of error. The case with Down syndrome and syndrome X-like developmental characteristics exhibited a DNAm age of 31.2 years which was 6 years older than her chronological age. The results for this DS subject are congruent with those from a recent study [16] that demonstrated that DS subjects exhibit accelerated epigenetic aging effects.

The small sample size did not provide sufficient statistical power for detecting any obvious abnormalities in the DNA methylation profiles. No statistically signific-ant epigenetic markers (CpGs) were found after correct-ing for multiple comparisons. None of the CpGs were significant at a false discovery rate threshold of 0.05.

## DISCUSSION

We identified five new cases whose clinical presentations were similar to the first case. Prior screening eliminated any of the recognized causes of developmental delay and selected subjects also retained youthful facial and structural/functional features atypical of children suffering growth retardation of known etiology. As with the first case, the newly discovered cases lacked any obvious genetic problems according to karyotype and a microarray comparative genome hybridization analysis. Thus, while extremely rare, the first case described was not unique in the world. Furthermore, since such children require extensive medical care to survive, especially during the first years after birth, it may be that most succumb before ever being diagnosed. While the protocol did not mandate gender selection/preference, all subjects were female. It is not known whether this occurrence was due to chance alone or is a sex linked aspect of the putative syndrome. According to the epigenetic clock method, blood tissue from these cases is age appropriate. These findings are consistent with leukocyte telomere measurements in our first case [1]. Incidentally, leukocyte telomere length captures another dimension of aging and is at best weakly correlated with DNAm age after correcting for chronological age [12, 13].

Our results demonstrate that despite the clinical appearance of delayed maturation in children afflicted with syndrome X, the epigenetic clock indicates that the rate of development in blood and perhaps other tissues is normal. Thus, while we cannot exclude tissue-specific ageing as causal in syndrome X, the current findings suggest that the observed delay in whole body development results from other, yet undiscovered factors.

Overall, our analysis of blood tissue shows that aging in our study subjects is not arrested in all tissues. Future studies should assess whether other tissue types from these subjects (or their bodies as a whole) evade epigenetic aging.

## METHODS

### DNA methylation data

The DNA methylation data set has been deposited in Gene Expression Omnibus (GSE64495).

Blood samples were drawn sequentially on a given day from all members of the same family into anticoagulant (EDTA) containing tubes, immediately frozen on dry ice and shipped overnight to the processing center. Tubes were coded to prevent bias in subsequent analysis. Genomic DNA was extracted and purified using the RecoverEase DNA Isolation Kit (Agilent Technologies, La Jolla, CA, USA). Bisulfite conversion using the Zymo EZ DNA Methylation Kit (ZymoResearch, Orange, CA, USA) as well as sub-sequent hybridization of the Human Methylation450K Bead Chip (Illumina, SanDiego, CA), and scanning (iScan, Illumina) were performed according to the manufacturers protocols by applying standard settings. DNA methylation levels (β values) were determined by calculating the ratio of intensities between methylated (signal A) and un-methylated (signal B) alleles. Specifically, the β value was calculated from the intensity of the methylated (M corresponding to signal A) and un-methylated (U corresponding to signal B) alleles, as the ratio of fluorescent signals β = Max(M,0)/[Max(M,0)+Max(U,0)+100]. Thus, β values ranged from 0 (completely un-methylated) to 1 (completely methylated). We used background corrected beta values that result from the BeadStudio software (version 3.2).

### Estimation of DNAm age using the epigenetic clock method

Statistically speaking, the epigenetic clock is a prediction method of age based on the DNAm levels of 353 CpGs dinucleotides. Predicted age, referred to as DNAm age, correlates with chronological age in sorted cell types (CD4 T cells, monocytes, B cells, glial cells, neurons) as well as in tissues and organs including whole blood, brain, breast, kidney, liver, lung, saliva [11]. Mathematical details and software tutorials for the epigenetic clock can be found in the Additional files of reference [11]. An online age calculator is provided at our webpage [21].
